# The Environmental “Riskscape” and Social Inequality: Implications for Explaining Maternal and Child Health Disparities

**DOI:** 10.1289/ehp.8930

**Published:** 2006-04-06

**Authors:** Rachel Morello-Frosch, Edmond D. Shenassa

**Affiliations:** 1 Center for Environmental Studies; 2 Department of Community Health, School of Medicine and; 3 Centers for Behavioral and Preventive Medicine, Brown University, Providence, Rhode Island, USA

**Keywords:** birth outcomes, environment, health disparities, stress

## Abstract

**Background:**

Research indicates that the double jeopardy of exposure to environmental hazards combined with place-based stressors is associated with maternal and child health (MCH) disparities.

**Objective and Discussion:**

Our aim is to present evidence that individual-level and place-based psychosocial stressors may compromise host resistance such that environmental pollutants would have adverse health effects at relatively lower doses, thus partially explaining MCH disparities, particularly poor birth outcomes. Allostatic load may be a physiologic mechanism behind the moderation of the toxic effect of environmental pollutants by social stressors. We propose a conceptual framework for holistic approaches to future MCH research that elucidates the interplay of psychosocial stressors and environmental hazards in order to better explain drivers of MCH disparities.

**Conclusion:**

Given the complexity of the link between environmental factors and MCH disparities, a holistic approach to future MCH research that seeks to untangle the double jeopardy of chronic stressors and environmental hazard exposures could help elucidate how the interplay of these factors shapes persistent racial and economic disparities in MCH.

A formidable challenge in the field of maternal and child health (MCH) has been explaining the persistent racial and socioeconomic health disparities, particularly in birth outcomes in the United States (Singh and Yu 1995). Despite declines in the overall infant mortality, there remains a significant disparity between black and white infant mortality rates. Nationally, black women are twice as likely as white women to give birth to a very low-birth-weight baby. For preterm births, although the gap between the two racial groups has narrowed recently, the disparity between the two groups remains large: a 6.7% difference (National Center for Health Statistics 2003).

Here we discuss the interplay of environmental hazards with place-based and individual-level psychosocial stressors and its implications for MCH research. Although a strong body of literature has shed much light on the individual-level factors (e.g., health behaviors, inter-pregnancy interval, and access to adequate health care) (Hessol et al. 1998; Hummer 1993; Rawlings et al. 1995; Starfield et al. 1991) and placed-based drivers of MCH disparities (e.g., neighborhood poverty, relative income inequality, poor housing, and segregation) (Buka et al. 2002; Guest et al. 1998; Huynh et al. 2005; Laveist 1993; Matteson et al. 1998; Morenoff 2003; O’Campo et al. 1997; Shenassa et al. 2004), there has been little cross-pollination between this field and the research investigating links between environmental hazard exposures and birth outcomes (Parker et al. 2005; Ritz and Yu 1999; Ritz et al. 2000; Sadler et al. 1999; Whyatt et al. 2004; Wilhelm and Ritz 2003).

Place-based stressors are biologically relevant components of the human environment and can function independently of individual-level stressors to determine health (Diez-Roux 1998; Shenassa 2001). These place-based factors can influence birth outcomes in three ways: *a*) by affecting birth outcomes directly (Huynh et al. 2005; Rich-Edwards and Grizzard 2005); *b*) by increasing exposures to environmental hazards, such as air pollutants (Parker et al. 2005; Woodruff et al. 2003); and *c*) by enhancing susceptibility to the toxic effects of contaminant exposures (Ponce et al. 2005). This third pathway concerning the interaction of place-based stressors with environmental hazards points toward the next generation of studies to understand the combined effects of environmental and psychosocial drivers of MCH disparities. We first discuss the confluence of place-based psychosocial stressors and environmental hazard exposures and its implications for future research on MCH disparities with a focus on birth outcomes. We then propose a possible physiologic link between place-based stressors and environmental hazards in ways that may enhance susceptibility to toxics. We conclude by outlining a conceptual framework for future MCH research.

## Social Inequality and Environmental Health Disparities

Wide-ranging political, socioeconomic, and discriminatory forces coupled with spatial patterns of industrialization and development have segregated people of color, particularly African Americans, into communities with some of the highest indices of urban poverty and material deprivation (Morello-Frosch and Jesdale 2006; Schultz et al. 2002; Williams and Collins 2004). Researchers and policy makers concerned about environmental justice argue that communities of color and the poor face a higher frequency and magnitude of exposures to environmental as well as psychosocial stressors [Institute of Medicine (IOM) 1999; O’Neill et al. 2003]. Concern has centered on the limited science related to the cumulative impact of multiple exposures to environmental hazards and the potential vulnerability of poor communities to their toxic effects [National Environmental Justice Advisory Council (NEJAC) 2004]. This combination and potential interaction of elevated environmental hazard exposures, on the one hand, and socioeconomic stressors, on the other, have been described as a form of “double jeopardy” (IOM 1999).

Understanding the MCH implications of these “geographies of exposure and susceptibility” (Jerrett and Finkelstein 2005) or “riskscapes” (Morello-Frosch et al. 2001) requires consideration of the timing of exposure to psychosocial stressors as well as environmental hazards during the life course (e.g., during the prenatal years, infancy, adolescence, or adulthood) and socioeconomic, political, cultural, and gender dynamics. For example, the lack of child care for agricultural workers often forces families, mostly mothers, to take their children to the fields while they work, thereby increasing young children’s exposures to pesticides (Natural Resources Defense Council 1999). Many of these pesticides are known neurotoxicants and carcinogens, and the potential long-term effects of childhood and prenatal exposures are just being explored and understood (Berkowitz et al. 2004; Castorina et al. 2003; Eskenazi et al. 2004; Gladen et al. 2003; Perera et al. 2003; Torres-Arreola et al. 2003; Whyatt et al. 2004; Young et al. 2005). Similarly, neighborhood-level factors associated with racial residential segregation may affect health by influencing access to affordable markets with fresh fruits and vegetables and access to health services (Diez-Roux 1997; Morland et al. 2002). Women without access to adequate prenatal care, for example, are likely to have compromised nutritional status, which in turn can heighten the impact of lead exposure both *in utero* and during early childhood (Lee et al. 2005; Zierold 2004).

## State of the Evidence

Research on birth outcomes points to the validity of integrating social with environmental health riskscapes in future MCH research (Gee and Payne-Sturges 2004; Morenoff 2003; O’Campo et al. 1997). Evidence shows a consistent relationship between residence in poverty-stricken (Collins et al. 1997; O’Campo et al. 1997; Papacek et al. 2002), segregated (Guest et al. 1998; Laveist 1993) neighborhoods and poor birth outcomes. Moreover, preliminary work suggests substantial racial and ethnic disparities in environmental hazards exposures (Centers for Disease Control and Prevention 2005; IOM 1999; Morello-Frosch 2002; NEJAC 2004), including during pregnancy (Woodruff et al. 2003), and studies have begun to link pollutant exposures and negative birth and developmental outcomes (Dejmek et al. 1999; Ritz and Yu 1999; Ritz et al. 2000, 2002). One recent study of individual factors, pollutant exposures, and neighborhood measures of socioeconomic hardship (Ponce et al. 2005) found that preterm birth risk was affected by the interaction of residential traffic-related air pollution exposure and measures of neighborhood economic hardship.

Distilling the results of this diverse body of MCH research reveals two critical paths for future inquiry. The first is the direct health effects of hazardous social and physical environments to which communities of color and the poor are disproportionately exposed. To date, MCH studies have emphasized this first line of inquiry by analyzing the effects of individual and place-based socioeconomic status (SES) stressors, on one hand, or by assessing the effect of individual factors and environmental hazards, on the other.

The second line of inquiry, as outlined below, examines all of these factors in an integrated fashion by exploring how the multilevel interplay and possible interaction of psychosocial stressors with environmental hazards may shape disparities in birth outcomes. For example, previous pollutant exposures may enhance susceptibility to the toxic effects of current pollutant exposures, particularly if the body’s defense mechanisms and ability to recover or detoxify have been compromised through prior exposures to harmful agents. Similarly, exposure to place-based psychosocial stressors, such as persistent poverty, material deprivation, and a lack of services, may lead to chronic stress, which can weaken the body’s defense systems (Cohen 1999; McEwen 1998).

## Physiologic Mechanisms

The concept of allostasis provides a framework for measuring the physiologic manifestations of chronic psychosocial and environmental stressors. Allostasis refers to the ability of the body’s stress–response systems to regulate internal physiology in response to psychosocial or physical stressors. The related concept of allostatic load refers to the cumulative physiologic degradation, over the life course, that can result from chronic stress exposure, and the accompanying long-term shift that occurs in the body’s homeostatic functions, with harmful consequences (Geronimus 1992; McEwen 1998; Seeman et al. 1997). The physiologic effect of prolonged stressors can exact a toll on the body that is caused by chronic activation of biologic systems, such as the hypothalamic–pituitary–adrenal axis, which releases hormones (e.g., glucocorticoids) that can have several metabolic and psychological effects, including the mobilization of energy reserves and suppression of the immune system (Seeman et al. 1997; Sterling and Eyer 1988). Chronic activation of this system can lead to “wear and tear” on major organ systems (McEwen 1998).

The mechanism of allostatic load provides a potential pathway by which place-based stressors can modify the toxic effect of environmental hazard exposures to produce disparate patterns of birth outcomes between and within populations. This is also in line with the concept of “weathering” proposed by Geronimus and others, suggesting that chronic stress associated with the combined effects of poverty, racial discrimination, and material deprivation causes the health of African-American mothers to deteriorate particularly rapidly, leading to poorer birth outcomes with increased age (Collins and Williams 1999; Geronimus 1992). The biomechanics of stress and allostasis can be considered a possible mediator of the heightened susceptibility to the adverse effects of pollution exposures observed among people with low SES.

## A Framework for Future Research

**Figure 1:**
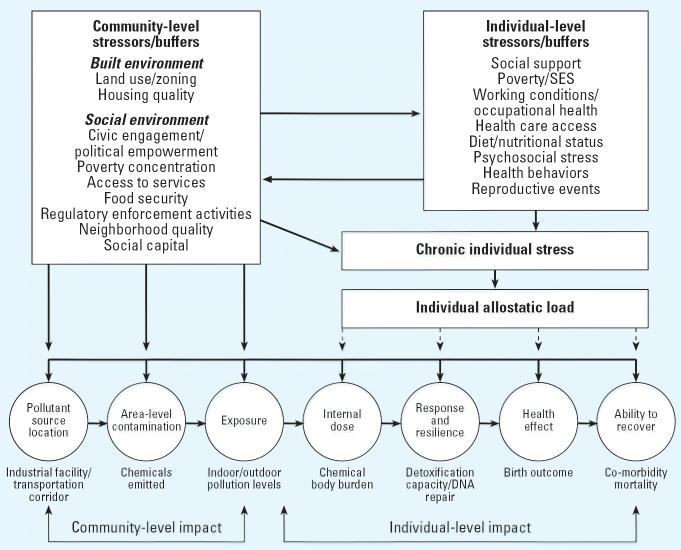
The interplay of community and individual stressors/buffers that shape exposures and susceptibility to environmental hazards. Thick arrows indicate relationships that have been studied in the epidemiologic and sociology literature; dashed arrows indicate relationships that have not been extensively explored.

The framework in Figure 1 suggests how area-level and individual-level stressors and buffers may combine to shape environmental hazard exposures, affect individual allostatic load, and in turn enhance susceptibility to the toxic effects of pollution exposures. At the bottom of Figure 1 is a variation of the exposure–health outcome continuum that outlines how environmental toxins might cause disease (National Research Council 1991). Traditionally, the exposure–health outcome continuum includes the emission of a contaminant from an indoor source such as smoking or an outdoor source such as an industrial facility, through human exposure via various media (e.g., air), and the occurrence of an adverse health outcome (e.g., low birth weight). The framework depicted in Figure 1 implies that the presence of an environmental contaminant must first lead to human exposure and then overcome the body’s defense systems to have an adverse health effect. The internal dose may not have an adverse health effect until it achieves a biologically effective dose that depends on rates of bioaccumulation, biotransformation, elimination, and, most relevant to our discussion, an individual’s susceptibility.

Animal studies suggest that stress can moderate a response to environmental toxins and other agents. For example, chronic stress may increase the absorption of environmental toxicants into the body through increased respiration, consumption, or perspiration (Gordon 2003). Similarly, allostatic load may amplify susceptibility to the toxic effects of pollutants, leading to adverse birth outcomes. Indeed, stress may alter physiologic functioning, through stress-dependent hormones that can affect *in utero* development and shift the threshold for toxicity, thereby leading to adverse birth outcomes at lower exposures (Paarlberg et al. 1995). Moreover, the body’s biotransformation or detoxification systems can remove or nullify toxins, but under conditions of chronic stress, the body’s defense system may be impaired, resulting in compromised organ resistance. Finally, illness caused by chronic stress may compromise a sick individual’s capacity to cope with environmental hazard exposures (Rios et al. 1993).

## Conclusion

Allostatic load may be a critical physiologic mechanism that explains the excess burden of adverse birth outcomes related to certain pollutants observed among low-SES populations and some communities of color. Maternal immune systems that are shaped by chronic stressors before conception and during pregnancy may enhance particular vulnerabilities to adverse pregnancy outcomes. This is compounded by race- and class-based disparities in exposures to environmental hazards that are driven by the distribution of power, privilege, and economic resources (Morello-Frosch 2002). These environmental health disparities are likely to be moderated by the degree and magnitude of chronic community and individual-level stressors that may be reflected in individuals’ allostatic load. Therefore, a holistic approach to future MCH research that seeks to untangle the double jeopardy of chronic stressors and environmental hazard exposures could help elucidate how the interplay of these individual- and community-level factors shape persistent racial and economic disparities in birth outcomes. Most important, for researchers and practitioners concerned about environmental justice, this line of inquiry could suggest new strategies for alleviating systemic drivers of racial and socioeconomic disparities in birth outcomes.
